# A Single-Connector Stent Antenna for Intravascular Monitoring Applications

**DOI:** 10.3390/s19214616

**Published:** 2019-10-23

**Authors:** Chien-Hao Liu, Shuo-Chih Chen, Hao-Ming Hsiao

**Affiliations:** Mechanical Engineering Department, National Taiwan University, Taipei 10617, Taiwan; r06522509@ntu.edu.tw (S.-C.C.); hmhsiao@ntu.edu.tw (H.-M.H.)

**Keywords:** smart stent, implantable antennas, vascular restenosis, blood pressure, blood flow velocity, PWV, FEM, intravascular monitoring

## Abstract

Recently, smart stents have been developed by integrating various sensors with intravascular stents for detecting vascular restenosis or monitoring intravascular biomedical conditions such as blood pressure or blood flow velocity. The information on biomedical signals is then transmitted to external monitoring systems via wireless communications. Due to the limited volumes of blood vessels and limited influence of blood flow, antennas with good radiation performance are required for intravascular applications. In this paper, we propose a stent antenna composed of multiple rings containing crowns and struts, where each ring is connected with one connector. Unlike a conventional stent, wherein each ring is connected with several connectors, the single connector prevents the random distribution of electrical current and thus achieves good radiation performance. The implantable stent antenna is designed for the frequency range of 2 to 3 GHz for minimum penetration loss in the human body and tissues. Mechanical FEM simulations were conducted to ensure that the mechanical deformation was within specific limits during balloon expansions. A prototype was fabricated with laser cutting techniques and its radiation performance experimentally characterized. It was demonstrated that the fabricated stent antenna had an omnidirectional radiation pattern for arbitrary receiving angles, a gain of 1.38 dBi, and a radiation efficiency of 74.5% at a resonant frequency of 2.07 GHz. The main contribution of this work was the manipulation of the current distributions of the stent for good EM radiation performances which needed to be further examined while inserted inside human bodies. These research results should contribute to the further development of implantable wireless communications and intravascular monitoring of biomedical signals such as blood pressure and blood flow velocity.

## 1. Introduction

In past decades, intravascular stents have been widely used in the medical treatment of vessel obstructions for patients with vascular diseases such as stroke, heart attack, and aneurysm [1,2]. Stents are essentially wire meshes inserted into narrow blood vessels via a catheter and medical surgery. The narrow parts of the blood vessels are expanded by balloons to restore regular blood flow. However, the long-term usage of stents inside the blood vessels leads to vascular restenosis (i.e., vessel re-narrowing) due to the growth of vascular smooth muscle cells [3]. The re-narrowing of blood vessels hinders the blood flow and can cause severe problems. These issues can be alleviated by eluting drugs [4,5], manipulating shear stresses on the vessel walls [6], or heating the re-narrowing regions to suppress the growth of vascular smooth muscle cells [7,8].

**Figure 1:**
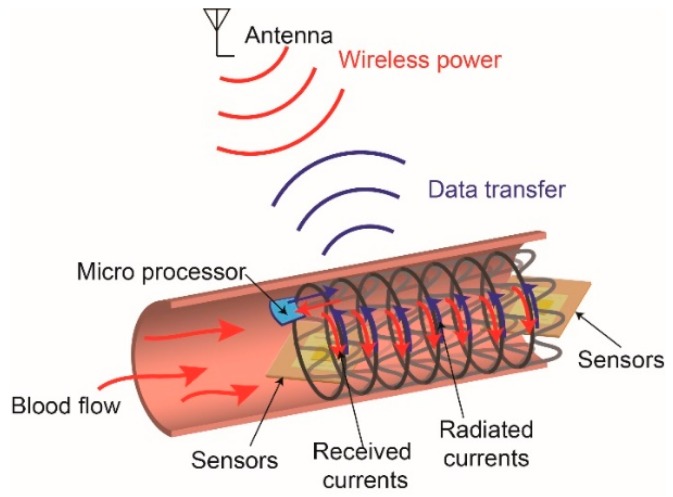
Illustration of a smart stent wherein the entire stent acts as an antenna for transferring biomedical information measured via integrated sensors to the external monitoring system.

For early diagnosis of restenosis and real-time monitoring of intravascular blood conditions, smart stents or intelligent stents have been developed by integrating versatile sensors and wireless electronic systems with stents, as shown in Figure 1 [9,10]. For example, pressure sensors are used to measure intravascular blood pressure [9,11,12,13,14,15,16], multiple pressure sensors are used to measure the pulse wave velocity (PWV) [17], flow sensors can provide blood flow velocity [2,18,19], and other embedded sensors are utilized to monitor glucose [20] and temperature [19]. In fact, measuring blood pressure intravascularly, directly from blood vessels, provides accurate and real-time values of blood pressure that are superior to those of non-invasive blood-pressure approaches such as ultrasound [21], the volume-clamp method [22]. These sensors mounted on the stents should have low profiles and small sizes to avoid potential obstacles for regular blood flows [12]. The measured intravascular signals can be signal-processed by embedded microprocessors or transmitted to external monitoring systems via wireless communications [11,23].

In general, intravascular stents are small in size, ranging in length from 12 to 38 mm and in diameter from 2 to 10 mm. Thus, it is necessary to miniaturize implantable electronic devices and communication systems. Various wireless power/data communications approaches for smart stents have been investigated, including passive LC-resonant antennas [7,8,9,12,17,24,25,26], miniaturized packaged antennas [20], RFIC [11], and active stent antennas [10,14,15,19,23]. LC-resonant antennas are composed of metallic stents acting as inductors and additional capacitors forming LC resonant circuits. Via strong magnetic field couplings with external loop antennas and the phase dip technique, the intravascular information can be obtained through the variations of the resonant frequencies. However, one disadvantage of LC resonant antennas is that the active regions of the transmitting loop antennas should be overlapped with the receiving inductive stents for strong magnetic-field couplings [17]. In some circumstances, it is difficult to locate coronary artery stents by the commonly used CT angiography [27] or MRI [28]. However, the whole stent can be considered as an antenna for receiving electromagnetic signals and power from transmitting antennas at arbitrary angles [23]. For stent antennas, the requirements that need to be met are long-term usage, biocompatibility, electrical conduction, and radial expansion capability, as well as structural integrity after expansion to prevent the influence of blood flow. 

In this paper, we propose a stent antenna for intravascular monitoring and implantable wireless applications. The stent antenna was expected to have better radiation performance than that of LC resonant antennas at low frequencies, such as 900 MHz [19]. Modern stents are composed of open cells, each unit is composed of crowns and struts, and each ring is connected by multiple connectors [29]. However, this design would cause random current distribution and reduce radiation performance. The goal of this work was to design a stent antenna with good radiation performance by using a single connector between each unit of the stent. The single-connector stent antenna eliminates the complicated and meandering geometry of a typical stent, which would cancel out the induced currents and be unable to excite the desired electromagnetic waves. In addition, a stent with single connectors should maintain a stable mechanical structure and not exceed fracture limits during balloon expansion [1]. The single-connector stent antenna could be applied to other vessels and not limited to coronary arteries.

This paper is organized as follows. In the next section, the design of the proposed stent antenna and its operation frequency are presented. Then, the EM and FEM simulations determining the electrical properties and mechanical FEM simulations for verifying the mechanical properties are described. Subsequently, the laser-cutting fabrication process and balloon expansions of prototypes are disclosed. Finally, the radiation characteristics of the fabricated stent antenna are measured in an anechoic chamber and discussed. Important results are summarized at the end of the paper.

## 2. Designs

**Figure 2:**
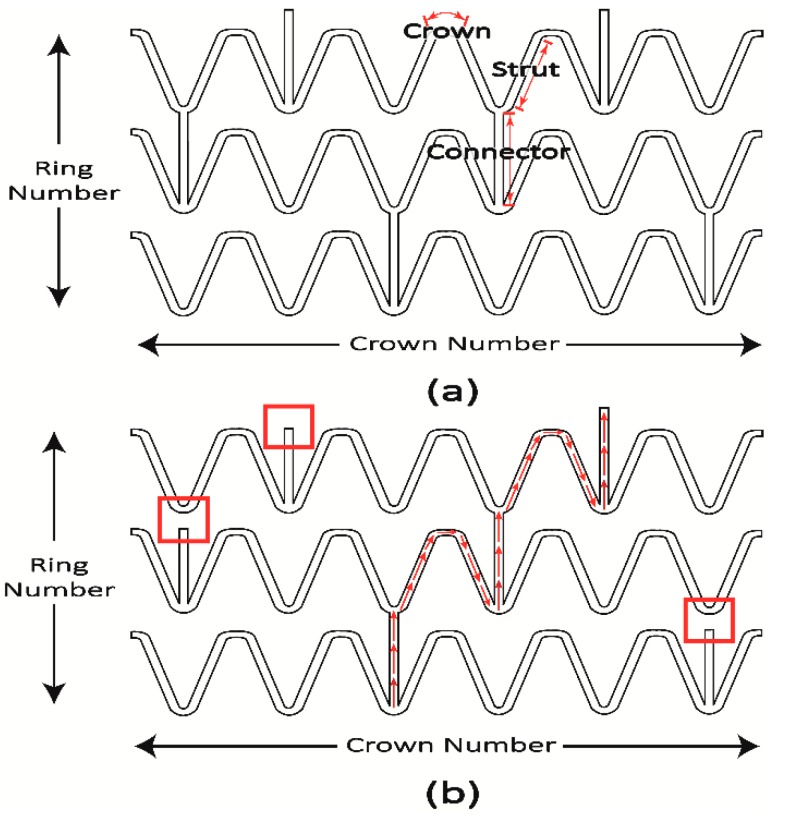
2D planar geometries of (**a**) multi-connector, and (**b**) single-connector stent antennas. The red boxes indicate that a connector is disconnected between two adjacent rings. The red arrows indicate the surface current flow path at the resonant frequency.

**Figure 3:**
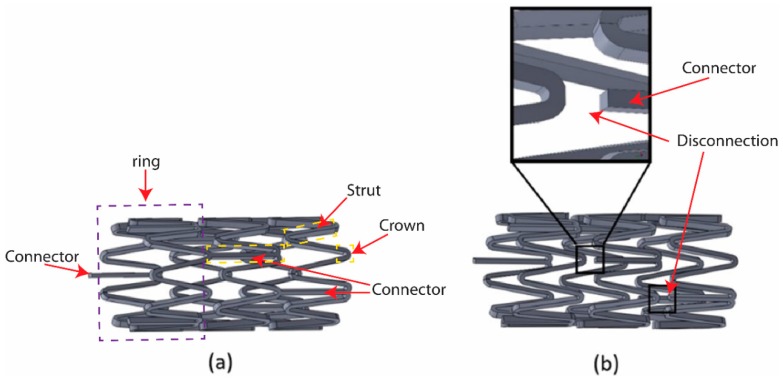
3D topologies of (**a**) multi-connector and (**b**) single-connector stent antennas.

Figure 2a presents the 2D unfolded planar geometry of a multi-connector stent. In general, multi-connector stents are composed of several rings connected by connectors with cylindrical shapes for providing sufficient radial force to open narrowed blood vessels [5,24]. Each ring consists of crowns and struts, and the crowns are circular so that they can expand and achieve large deformation under balloon expansions. Most commercially available multi-connector stents have two to three connectors between adjacent rings to achieve the desired structural stability [30,31]. However, these multiple connectors can lead to random current distributions and are not suitable for antenna applications. In this paper, we propose a single-connector stent antenna having adjacent rings connected by a single connector, as shown in Figure 2b. A single-connector stent antenna can allow current distribution with an approximate half-wavelength resonant mode. The current distributions will be discussed in the next section. Figure 3a,b presents the 3D geometries of a multi-connector and the proposed single-connector stent antennas where undesired connectors are not connected.

**Figure 4:**
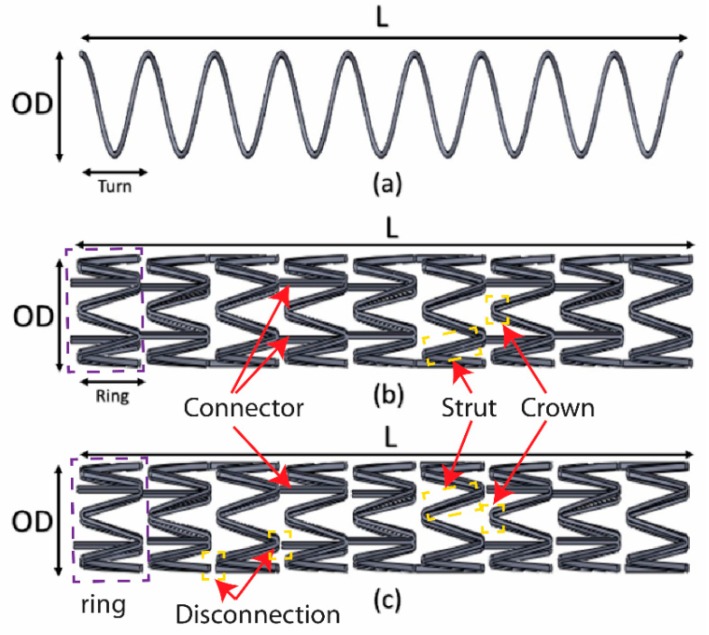
3D topologies of the final design of (**a**) helical stent, (**b**) regular multi-connector stent, and (**c**) single-connector stent.

For implantable antennas, the resonant frequency of the stent antenna depends on the total length of the stent and its geometry. In fact, in the human body, electromagnetic waves (EM) have different penetration attenuations and variable power loss boundaries at different frequencies [23,32]. For low-frequency electromagnetic coupling communications, the optimal operation frequency is in the MHz range for low tissue absorption loss where the biological tissues are modeled as dispersive medium and results in large sizes of implanted antennas [23]. For high-frequency wireless communications, the human body is modeled as a low-loss multi-layer dielectric medium and the optimal frequency is in the range from 2 to 3 GHz for the minimum tissue absorptions and good orientation tolerances of implanted antennas [32]. Therefore, the single-connector stent antenna was designed to have a resonant frequency in the range of 2 to 3 GHz that the total length of the surface current flow path shown in Figure 2b was close to a half wavelength expressed as

(1) $$\left(\mathrm{Strut}\;\mathrm{Length}+\mathrm{Crown}\;\mathrm{Length}+\mathrm{Connector}\;\mathrm{Length}\right)\times \left(\mathrm{Ring}\;\mathrm{Number}\right)≅\frac{\lambda }{2}$$

where λ was the wavelength of the first resonance. The strut length, crown length, and connector length are the lengths of the strut, crown, and connector shown in Figure 2. Furthermore, the length should be in the range of 10 to 20 mm, and the diameter, in the range of 2 to 10 mm due to the sizes of vessels [7,8]. In addition, this frequency range falls in the range of the ISM band, and a high operation frequency is beneficial for implantable antennas [23]. The final dimensions of the single-connector stent antenna were a length of 18 mm, 6 crowns, 9 rings, and a final diameter of 2 mm when inserted into blood vessels, as shown in Figure 4 and Table 1. For comparison, a multi-connector stent antenna and an old helix-shaped stent antenna of the same size were also investigated. The helical stent antenna had 9 turns within the same total length of 18 mm.

In this research, we employed the commonly used L-605 Cobalt-Chromium (Co-Cr) alloy as the material for all the stents because Co-Cr alloy has a smaller minimum thickness than conventional stainless steel [1]. As a ductile material, Co-Cr alloy has a Young’s modulus of 203 GPa, a Poisson’s ratio of 0.3, yield stress of 590 MPa, and ultimate strength of 1689 MPa.

## 3. Simulations

### 3.1. EM FEM Simulations

**Figure 5:**
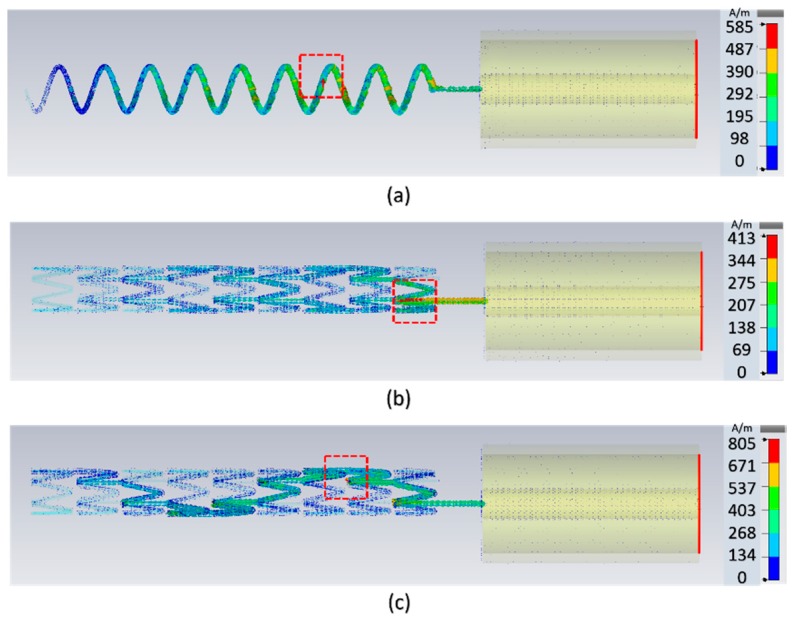
The simulated surface current distributions of the (**a**) helical stent, (**b**) multi-connector stent, and (**c**) single-connector stent antennas. At the resonant frequency, the multi-connector stent antenna has a radon current path where the helical and single-connector stent antennas have half-wavelength resonant modes. The red dashed box indicates the locations of the maximum current.

**Figure 6:**
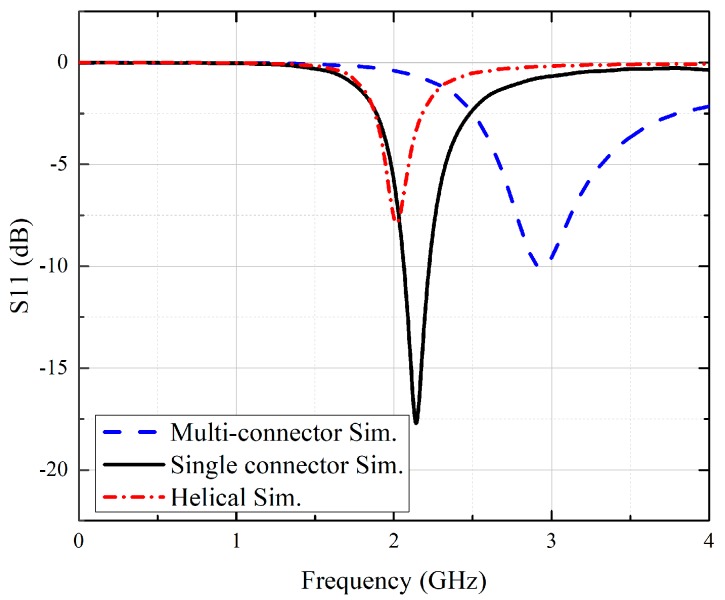
Simulated reflection coefficients of the helical stent, multi-connector stent, and single-connector stent antennas.

To investigate the EM properties of stent antennas, full-wave EM simulations were conducted to study the surface currents and radiation patterns with commercial software, CST Microwave Studio. Figure 5 shows the surface current distributions at the resonant frequency of the helical, multi-connector, and single-connector stent antennas. Most stent antennas had an issue of small ground planes due to limited small volumes of blood vessels [10,23]. Similarly, we used SMA connectors with finite ground planes for power extractions. It could be improved by combining two stent antennas to form a dipole-like antenna [23]. The multi-connector stent antenna had three connectors, and the random current flowed throughout the entire stent. In the single-connector stent antenna, there was a major current flow path between each ring, resulting in an approximate half-wavelength resonant mode similar to that of the helical stent antenna with a relatively small ground plane. Therefore, the single-connector stent antenna and the helical stent antenna had similar resonant frequencies, as can be observed from the simulated reflection coefficients in Figure 6. Since the multi-connector stent antenna had random current distribution, it had a higher resonant frequency with the same dimensions. This result supported that a single-connector stent antenna is suitable for stent antennas. 

### 3.2. Mechanical FEM Simulations

**Figure 7:**
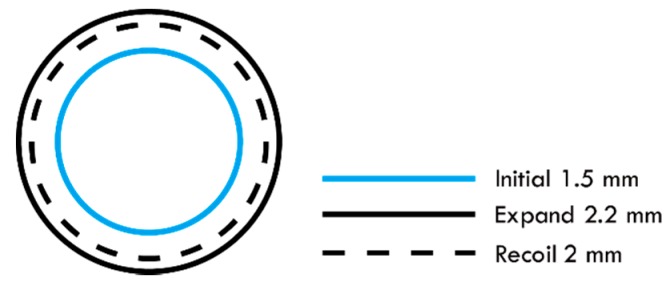
The cross-sectional view of the cylindrical stent for simulating the expansion process, including the initial state, the expansion due to the balloon, and the radial force exerted by the blood vessel.

**Figure 8:**
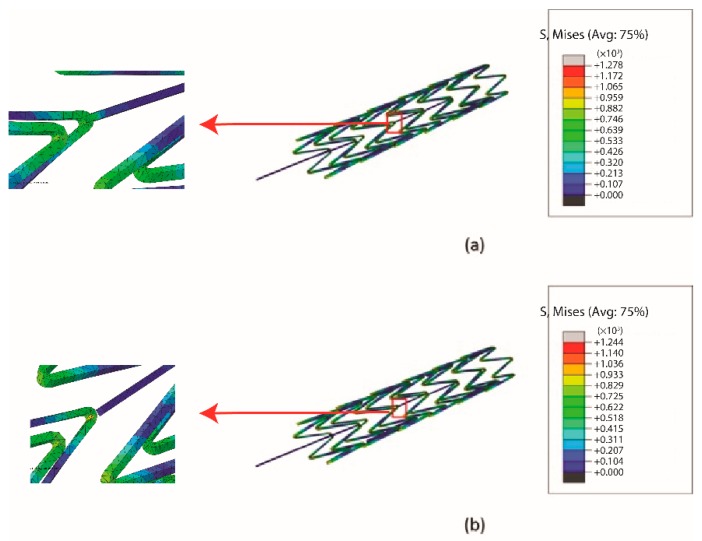
The simulated Von-Mises stress distributions of the (**a**) multi-connector, and (**b**) single-connector stent antennas via mechanical FEM simulations.

**Figure 9:**
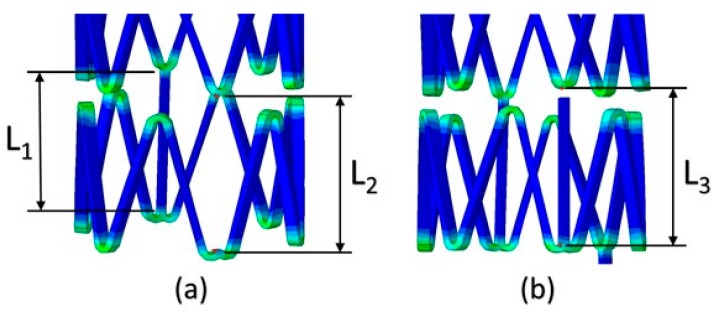
The comparisons of the single-connector stent antennas when the connector was (**a**) completely removed, and (**b**) partially removed.

To verify that the mechanical properties of the stent antennas fulfilled the requirements, mechanical finite element simulations were conducted in commercial software, ABAQUS (Dassault System Simulia Corp., Velizy Villacoublay, France). In accordance with the practical balloon expansion processes of stents, a cylindrical stent with an initial diameter of 1.5 mm was expanded to a diameter of 2.2 mm to simulate balloon expansion, as shown in Figure 7 [33]. Then the oversized stent was compressed to the desired diameter of 2 mm to simulate the radial force of the blood vessel walls. The simulated Von-Mises stress distributions of the multi-connector and single-connector stents are shown in Figure 8. In general, coronary stents use ductile materials that can achieve large deformation during balloon expansion [34]. They obey the Von-Mises stress criterion, expressed as

(2) $$\sigma_{von-mises}=\frac{{\left[{\left(\sigma_{1}-\sigma_{2}\right)}^{2}+{\left(\sigma_{2}-\sigma_{3}\right)}^{2}+{\left(\sigma_{3}-\sigma_{1}\right)}^{2}\right]}^{\frac{1}{2}}}{\sqrt{2}}\leq \sigma_{y}$$

where $\sigma_{1}$, $\sigma_{2}$, and $\sigma_{3}$ are principle stresses. The Von-Mises stress criterion indicates that ductile stents may fracture if the maximum Von-Mises stress exceeds the ultimate strength. The maximum Von-Mises stresses of the multi-connector and single connector stents in this research were lower than the ultimate strength of 1689 MPa of Co-Cr alloy. In other words, both stents had suitable mechanical properties for balloon expansions. In addition, the effect of removing the connector was described in Figure 9. If the connector was completely removed, it caused a longer longitudinal deformation, L2, than the original length of the connector, L1, resulting in undesired longitudinal elongations. This could be modified via partially removing the connector shown in Figure 9b that the deformed length, L3, was shorter than L2. Therefore, the trade-off design of the single connector was to sacrifice the longitudinal elongation and gain a better radiation efficiency.

## 4. Fabrication

**Figure 10:**
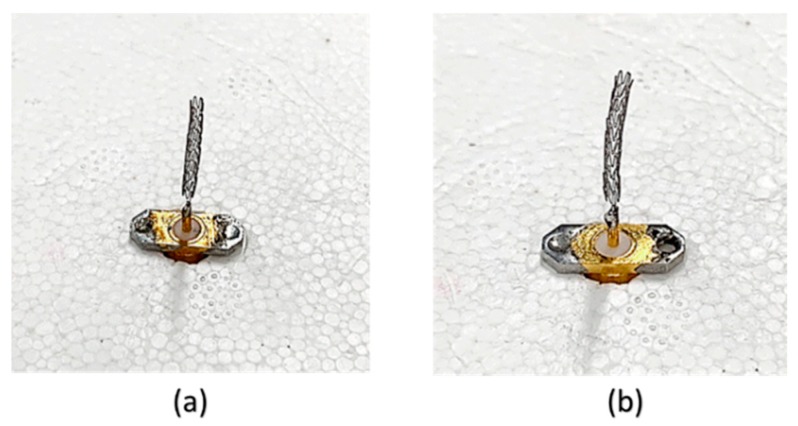
Photographs of the fabricated (**a**) multi-connector stent, and (**b**) single-connector stent placed vertically.

**Figure 11:**
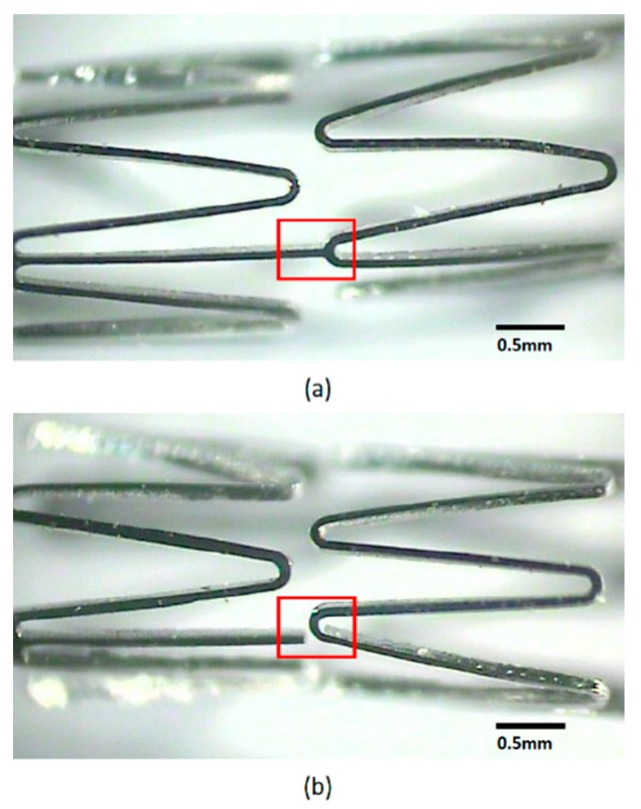
High-resolution photographs of the (**a**) multi-connector, and (**b**) single-connector stent antennas taken under an optical microscope.

Two prototypes of single-connector and multi-connector stents were fabricated via laser cutting of cylindrical Co-Cr alloy hypotubes. To pattern 2D unfolded geometries on the surfaces of the hypotubes, a 30W Nd-YAG pulse type fiber optical laser (Rofin) and an XY movement platform (Aerotech position) with precise position controls were employed. The details of the fabrication processes are reported in [1]. Figure 10 presents photographs of the fabricated multi-connector and single-connector stent antennas connected with SMA connectors for measurements. High-resolution photographs of the fabricated multi-connector and single-connector stent antennas viewed under an optical microscope are shown in Figure 11. In the single-connector stent antenna, each ring was connected via a single connector, and undesired connectors were not connected, as highlighted with a red box.

## 5. Measurements

### 5.1. Reflection Coefficients

**Figure 12:**
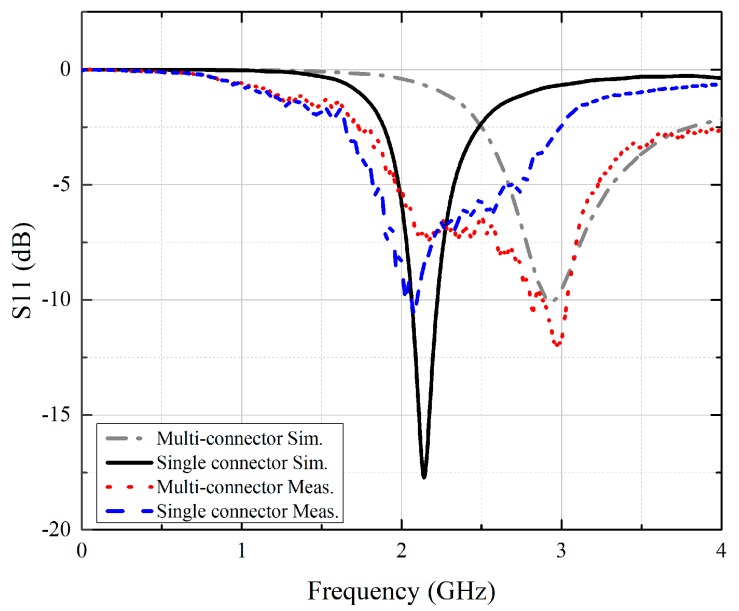
The simulated and measured reflection coefficients of the multi-connector and single-connector stent antennas.

The reflection coefficients of the fabricated single-connector and multi-connector stent antennas were measured with a calibrated vector network analyzer (Agilent E5071C). To reduce the effect of the feed line, an RF choke (Jin Hua Electronic Co., LTD, model: 2030000001866, Taipei, Taiwan) was utilized during the measurements. Figure 12 shows the simulated and measured reflection coefficients of both stent antennas. The resonant frequency of the single-connector stent antenna was lower than that of the multi-connector stent antenna of the same length. The simulation results matched the measured results, and the slight discrepancy was due to fabrication errors and tolerances.

### 5.2. Far-field Radiation Characteristics

**Figure 13:**
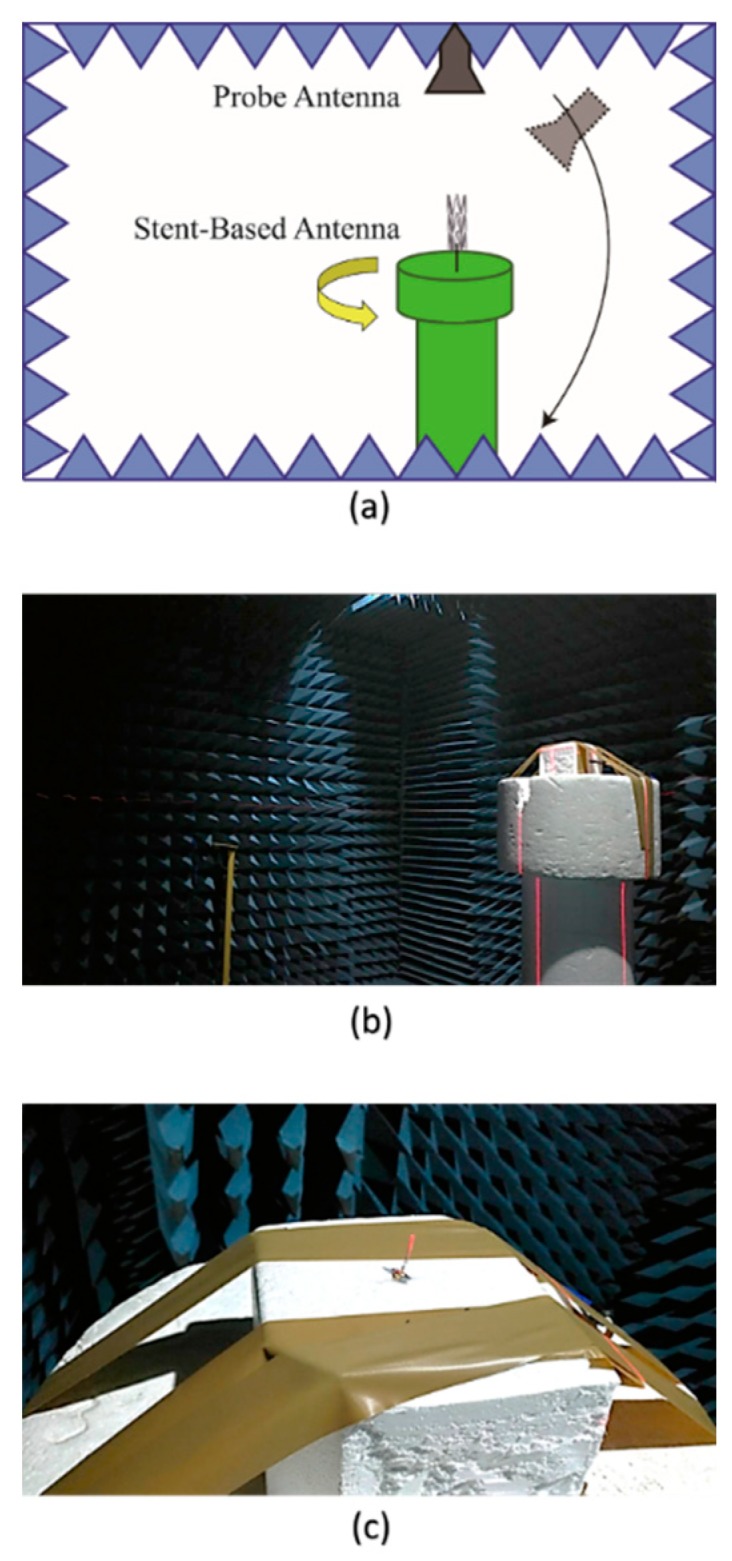
(**a**) Illustration of the measurement setup for 3D far-field radiation measurements, (**b**) The anechoic chamber, (**c**) The stent antenna mounted on a rotating stage to act as a receiving antenna.

**Figure 14:**
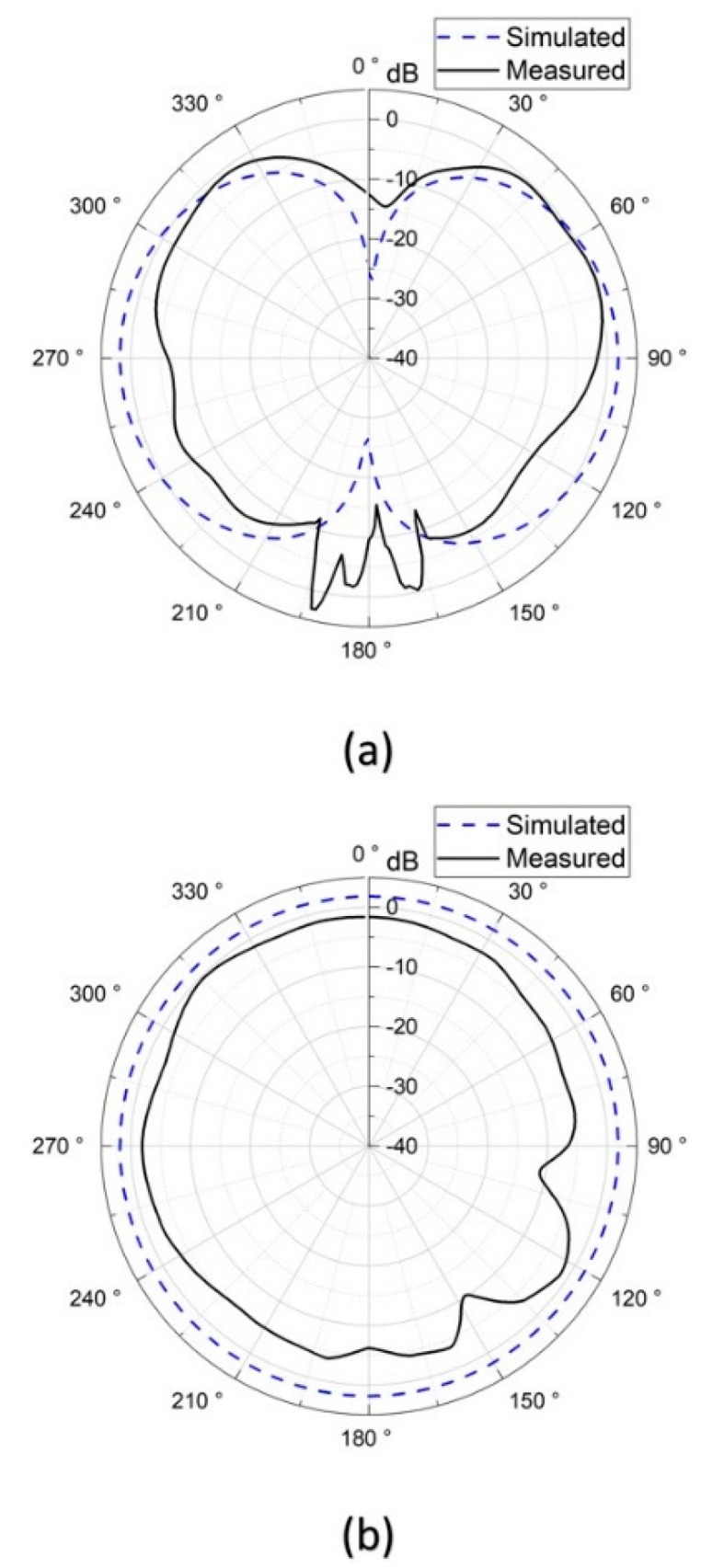
The gain patterns of the multi-connector stent antenna in (**a**) the E-plane, and (**b**) the H-plane.

**Figure 15:**
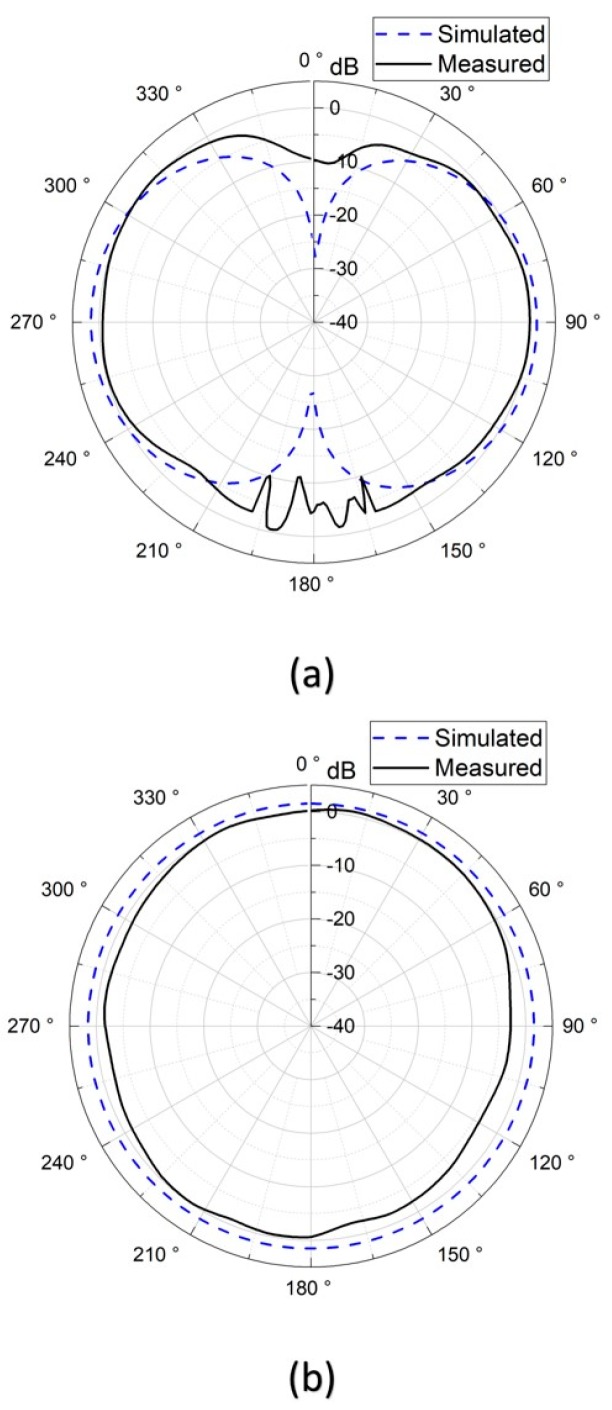
The gain patterns of the single-connector stent antenna in (**a**) the E-plane, and (**b**) the H-plane.

The 3D far-field radiation patterns were measured in the anechoic chamber at the Joint Laboratory for Microwave and Millimeter Wave Antenna Measurements at National Taiwan University of Science and Technology. Figure 13 shows the measurement setup. The stent antenna was mounted on a rotating stage and acted as a receiving antenna, and the probe antenna, which acted as a transmitting antenna, moved in the azimuth and elevation planes. The measured gain radiation patterns are shown in Figure 14 and Figure 15. Note that the measured radiation patterns at 180° were inaccurate due to the measurement setup where the transmitting antenna couldn’t move to the position underneath the stent antenna.

**Figure 16:**
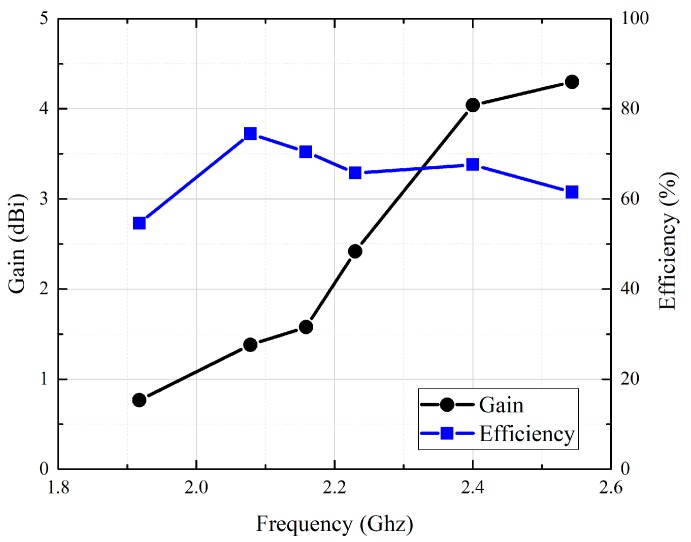
The measured gain and radiation efficiency of the single-connector stent antenna.

Figure 14a,b shows the simulated and measured gain radiation patterns of the multi-connector stent antenna at a resonant frequency of 2.97 GHz in the E- and H-planes, respectively. Similarly, Figure 15a,b shows the simulated and measured gain radiation patterns of the single-connector stent antenna at a resonant frequency of 2.07 GHz in the E- and H-planes, respectively. It can be seen that the radiation efficiency of the single-connector stent antenna was superior to that of the multi-resonant stent antenna due to its approximate half-wavelength resonant mode shown in Table 2. Although the multi-connector stent had a larger gain compared to that of the single-connector stent, both stent-based antennas had donut-shaped radiation patterns with omnidirectional radiation in the azimuth plane, which would be suitable for arbitrary receiving angles. Figure 16 shows the gain and the radiation efficiency of the single-connector stent antenna at different frequencies. It was demonstrated that the highest radiation efficiency of the single-connector stent antenna was 74.5% at the resonant frequency of 2.07 GHz, with a corresponding gain of 1.38 dBi. It indicated that a single-connector stent could provide the maximum radiation efficiency and omnidirectional electromagnetic emissions for intravascular wireless communications.

## 6. Discussions

In this research, we proposed a single-connector stent antenna composed of the stent and SMA connectors for good radiation efficiencies. The SMA connector acted as a finite ground plane. The simulation results showed that the entire stent antenna had a half-wavelength current distribution shown in Figure 5 and a dipole-link radiation pattern shown in Figure 14. For all measurements conducted in this research, an RF choke was exploited to reduce the effects of the feed line and only considered the stent antenna. The measured radiation pattern demonstrated a dipole-link radiation pattern as expected shown in Figure 14. The discrepancy between the simulated and measured radiation patterns at 180° was due to the measuring equipment that the receiving antenna cannot move underneath the stent antenna and cannot measure the EM radiation at 180°.

## 7. Conclusions

In this paper, we present a stent antenna for intravascular monitoring and implantable wireless applications that employ the entire stent as an antenna. The main contribution of this work was the manipulation of the current distributions of the stent for good radiation performance with a short stent length by exploiting a single connector between each unit of the stent. Unlike a general stent, wherein each unit is connected with multiple connectors, our proposed stent can achieve good EM radiation, maintain good mechanical strength, and remain within fracture limits during balloon expansions. A prototype was designed, fabricated, and experimentally examined with an EM anechoic chamber. The fabricated stent antenna demonstrated an omnidirectional radiation pattern, a gain of 1.38 dBi, and a radiation efficiency of 74.5% at the resonant frequency of 2.07 GHz. The results of this research should contribute to the development of implantable wireless communications and intravascular monitoring of cardiovascular diseases.

## Figures and Tables

**Table 1:** The geometric parameters of three stent antennas.

Stent Antenna	L (mm)	OD (mm)	Crown Number	Ring Number	Turn Number
Single	18	2	6	9	×
Multi	18	2	6	9	×
Helix	18	2	×	×	9

**Table 2:** The radiation characteristics of the stent antennas.

Stent antenna	Gain (dBi)	Directivity (dBi)	Efficiency (%)
Multi	4.94	6.527	69.5
Single	1.38	2.661	74.5
